# Spin-glass model predicts metastable brain states that diminish in anesthesia

**DOI:** 10.3389/fnsys.2014.00234

**Published:** 2014-12-11

**Authors:** Anthony G. Hudetz, Colin J. Humphries, Jeffrey R. Binder

**Affiliations:** ^1^Department of Anesthesiology, Medical College of WisconsinMilwaukee, WI, USA; ^2^Department of Neurology, Medical College of WisconsinMilwaukee, WI, USA

**Keywords:** anesthesia, consciousness, information, criticality, metastability, fMRI, resting state, functional connectivity

## Abstract

Patterns of resting state connectivity change dynamically and may represent modes of cognitive information processing. The diversity of connectivity patterns (global brain states) reflects the information capacity of the brain and determines the state of consciousness. In this work, computer simulation was used to explore the repertoire of global brain states as a function of cortical activation level. We implemented a modified spin glass model to describe UP/DOWN state transitions of neuronal populations at a mesoscopic scale based on resting state BOLD fMRI data. Resting state fMRI was recorded in 20 participants and mapped to 10,000 cortical regions (sites) defined on a group-aligned cortical surface map. Each site represented the population activity of a ~20 mm^2^ area of the cortex. Cross-correlation matrices of the mapped BOLD time courses of the set of sites were calculated and averaged across subjects. In the model, each cortical site was allowed to interact with the 16 other sites that had the highest pair-wise correlation values. All sites stochastically transitioned between UP and DOWN states under the net influence of their 16 pairs. The probability of local state transitions was controlled by a single parameter T corresponding to the level of global cortical activation. To estimate the number of distinct global states, first we ran 10,000 simulations at T = 0. Simulations were started from random configurations that converged to one of several distinct patterns. Using hierarchical clustering, at 99% similarity, close to 300 distinct states were found. At intermediate T, metastable state configurations were formed suggesting critical behavior with a sharp increase in the number of metastable states at an optimal T. Both reduced activation (anesthesia, sleep) and increased activation (hyper-activation) moved the system away from equilibrium, presumably incompatible with conscious mentation. During equilibrium, the diversity of large-scale brain states was maximum, compatible with maximum information capacity—a presumed condition of consciousness.

## Introduction

Cognitive functioning of the conscious human brain is thought to depend on the formation of dynamic patterns of neuronal coalitions and large-scale connectivity (Werner, 2009; Bressler and Menon, 2010). Moreover, the diversity or *repertoire* of distinct functional patterns reflects the information capacity of the brain that is thought to be central to consciousness (Tononi, 2008; Deco et al., 2014). The repertoire of brain states over time can be large if there is sufficient flexibility in the system to rapidly switch to new configurations and maintain these configurations for a finite amount of time. The time necessary for maintaining a configuration should roughly coincide with the duration of a conscious perceptual frame (Bachmann, 2013). The dynamic nature of the ongoing stream of consciousness may reflect this rapid sequence of state configurations (Werner, 2007). Moreover, the disruption of the sequence of states may account for the anesthetic suppression of consciousness (Hudetz et al., 2014).

Various physical, chemical, and biological systems are able to produce *metastable states* that satisfy the requirement for a large repertoire. Metastable states typically arise in critical systems that operate at the border of order and disorder and are characterized by complex patterns of fluctuations (Werner, 2007; Beggs, 2008; Kitzbichler et al., 2009; Deco and Jirsa, 2012; Tagliazucchi et al., 2012). Self-organization is often the underlying mechanism of criticality. Recent computational and empirical studies based on electrophysiology, fMRI, and EEG lend support to the existence of this behavior in the brain (Friston, 1997; Freeman and Holmes, 2005; Werner, 2007; Kitzbichler et al., 2009; Kelso, 2012; Bhowmik and Shanahan, 2013; Tognoli and Kelso, 2014). This metastability is considered essential to the subjective mental state and consciousness (Kitzbichler et al., 2009). Its restoration may be the hallmark of recovery from unconsciousness (Hudson et al., 2014).

Self-organization can also lead to scale-free behavior, in which similar interactions are present at different temporal or spatial scales (Tognoli and Kelso, 2014). The scaling of the magnitude of interactions is typically 1/f, where f is the frequency (Kello et al., 2008). Interestingly, the scale-free property of EEG or fMRI BOLD signals is preserved under anesthesia (Lee et al., 2010; Liu et al., 2014) but not in disorders of consciousness associated with diffuse brain damage (Liu et al., 2014). In light of this dissociation, conscious information processing may depend more closely on the dynamic repertoire and metastability of brain states than their spatio-temporal scaling laws.

Recent fMRI investigations have convincingly demonstrated that brain networks undergo dynamic reconfigurations even in the absence of novel stimuli or cognitive tasks (Britz et al., 2010; Chang and Glover, 2010; Sakoglu et al., 2010; Kang et al., 2011; Allen et al., 2012; Glerean et al., 2012; Handwerker et al., 2012; Jones et al., 2012; Cribben et al., 2013; Di and Biswal, 2013; Hutchison et al., 2013b; Keilholz et al., 2013; Liu and Duyn, 2013). Such resting state network dynamics have been ascribed to the general phenomena of spontaneous mentation, imagery, task-independent thoughts or daydreaming (Mckiernan et al., 2006).

The standard method for characterizing dynamic networks of the brain has been the sliding window analysis of functional connectivity (Hutchison et al., 2013a), sometimes combined with independent component analysis (ICA) (Kiviniemi et al., 2011), temporal ICA (Smith et al., 2012), and other source separation methods (Cribben et al., 2013). Connectivity analysis at higher temporal resolution has also been attempted with various point-process methods (Tagliazucchi et al., 2011), revealing so-called spontaneous co-activation patterns (Liu and Duyn, 2013; Liu et al., 2013). In all cases, a main limiting factor is the duration of the fMRI scan, which limits the number of connectivity patterns that can be extracted from a finite sample. On the other hand, the limited spatial resolution of EEG does not allow the imaging of spatially complex patterns. Moreover, collecting a sufficient amount of experimental data across the full range of brain states in the same subject, including multiple states of sleep, wakefulness, anesthesia, etc., is difficult.

As an alternative approach to explore the probability space of correlated brain states, we used a combination of empirical connectivity data and computer simulation. In the simulation, functional connectivity patterns were evolved by simulating the dynamic interaction of mesoscopic brain regions using a modified spin-glass model. This model is well-suited to describe the large-scale, globally distributed effect of the dynamic interaction of functionally connected brain regions. The model is relatively simple, as it does not include cell-specific or synaptic connections but it is minimally sufficient to account for an arbitrary pattern of neuronal interactions of distant, mesoscopic brain regions. Moreover, the model includes a single parameter to control the general cortical arousal level analogous in physical systems to the absolute temperature that determines the probability of spin fluctuations. The biological equivalent of spins in our model is the UP and DOWN states of neuronal activity.

As a novel feature, our model was constrained by using empirically derived resting state connectivity to set the spatial pattern of long-range interactions. The present approach is similar to the previously described Ising model (Fraiman et al., 2009; Das et al., 2014; Marinazzo et al., 2014), with the exception that our model is based on empirically derived long-range interactions. We show that with the chosen constraints set by the connectivity matrix, the model predicts critical behavior at the optimal activation level at which metastable states occur. Both reduced activation (anesthesia, sleep) and increased activation (epilepsy) moves the system away from equilibrium, presumably incompatible with conscious mentation. In equilibrium, the diversity of large-scale brain states is maximum, implying maximum information capacity—a previously postulated prerequisite of consciousness according to the Information Integration Theory (Tononi, 2008).

## Materials and methods

### fMRI experiments and data analysis

Resting-state BOLD fMRI data were collected from 20 subjects. Subjects were instructed to lie still with eyes open and avoid falling asleep. After each run, they were requested to rate their alertness level during the previous run. All imaging procedures were conducted on a 3.0 Tesla GE Excite scanner. For each subject, an anatomical scan was acquired using an SPGR pulse sequence (130 axial slices, slice thickness = 1.0 mm, TE = 3.2 ms, TR = 8.2 s, flip angle = 12 degrees, FOV = 240 × 180 mm, matrix size = 256 × 224). Resting state functional images were obtained using gradient-EPI (41 axial slices, slice thickness = 2.5 mm, TE = 25 ms, TR = 3 s, flip angle = 84°, FOV = 240 mm, matrix size = 96 × 96). In each subject, we obtained 6 runs of 7 min each (140 time points per run) for a total of 42 min of resting data.

Preprocessing of functional images included slice-timing correction, motion correction, and co-registration with the anatomical scan. To remove the effects of signal drift and possible artifacts due to motion, a regression analysis was conducted with third-order polynomial, the parameters from the motion correction algorithm, and a global signal regressor. These steps were performed using the software Analysis of Functional Neuroimages (AFNI, NIH). Cortical surface models were created from the anatomical scan of each subject using Freesurfer software. The subjects' surface model was aligned to an average surface atlas (FS Average brain in Freesurfer) using spherical surface-based alignment (Fischl et al., 1999). The triangular mesh of the FS Average brain was subsampled to 10,000 vertices across both hemispheres (Matlab reducepatch). The BOLD time courses were mapped to the 10,000 points by averaging all voxels overlapping with the cortical surface nearest to each of the 10,000 points. Each of the 10,000 points represents the average activity within a ~20 mm^2^ diameter patch of cortex. For spatial smoothing, an estimate of the distance between each of the points along the cortical surface was calculated by the fast marching algorithm (Sethian, 1996). Finally, correlation values were calculated between all pair-wise combinations of the 10,000 points. The resulting correlation matrices were averaged across subjects to create a single group connectivity matrix used for the simulation.

### Spin-glass model

The model was based on the standard assumption that global brain states evolve due to the ongoing interaction of mesoscopic brain regions, from here on called *sites*. The size of these sites was taken as that of the cortical surface patches of 20 mm^2^ from the fMRI data, each representing a vertical slab or macro-column of cortex. According to the standard Ising or spin-glass models, at any given time, each of the sites was assumed to be in one of two local states: UP (active) or DOWN (inactive). Further detailed description of the Ising model is available in previous publications (Fraiman et al., 2009; Kitzbichler et al., 2009; Das et al., 2014; Marinazzo et al., 2014). The essential difference between the Ising and spin-glass models is that the latter includes long-range interactions and variable interaction probabilities. Following the Monte-Carlo implementation of the Metropolis algorithm (Metropolis et al., 1953; Fricke, 2006), local states were allowed to flip with probability p_i_ as

$$\begin{array}{l} \;{\text{p}}_{\text{i}}\sim \exp\left(-\Delta {\text{E}}_{\text{i}}/\text{T}\right)\\ \Delta {\text{E}}_{\text{i}}=\frac{1}{2}{\text{S}}_{\text{i}}\sum_{\text{k = 1}}^{\text{n}}{\text{A}}_{\text{ik}}{\text{S}}_{\text{k}}\\ \;{\text{S}}_{\text{i}}=\left[+\text{1},\;-\text{1}\right],\text{ i}=\text{1},..,\text{n},\text{ k}\neq \text{i} \end{array}$$

In these equations, i and k index the cortical sites such that k is the index of sites interacting with site i, n is the total number of sites in the model, and A_ik_ is the connection matrix that defines the interacting sites. For each site i, S_i_ is the state variable (UP or DOWN), ΔE_i_ is the activation energy, and T is the global activation level—analogous to temperature in the physical literature.

To define the interacting sites, we used the fMRI functional connectivity data. For each site as a reference, the 16 other sites with the strongest correlation with the reference site were identified from the all pair-wise BOLD signal correlation matrix. Each site was then allowed to interact with their 16 pairs at a probability determined by the site's activation energy and the overall activation level.

In various runs, the activation level T was varied from zero to 4.0. Low values of T were taken as corresponding to reduced activation, such as in sleep, anesthesia or coma, and high values of T were taken as corresponding to hyper-activation, as in seizure. Each simulation started with a random distribution of UP/DOWN states as an initial condition. The system was then allowed to evolve for 10,000 time steps. Depending on the chosen value of T, the states converged or continued to change until the simulation was terminated.

## Results

### BOLD functional connectivity

**Figure 1:**
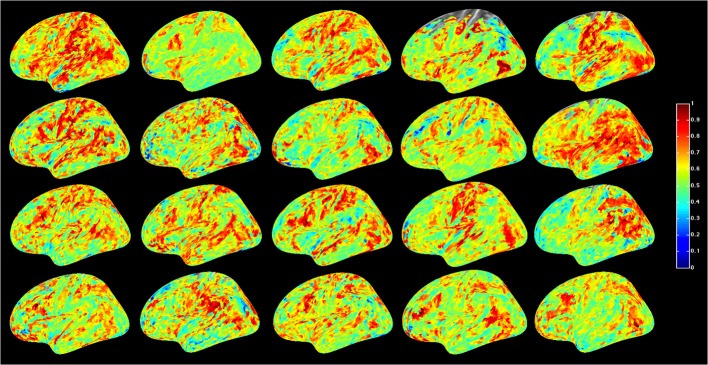
**Global resting-state cortical correlation maps in 20 subjects**. Pseudo-color indicates the average cross-correlation of each voxel with the rest of the brain.

**Figure 2:**
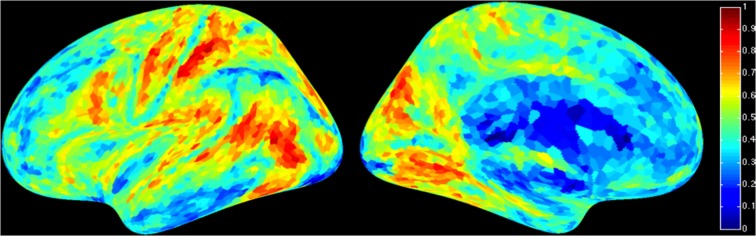
**Average global resting-state cortical correlation map from 20 subjects**. Pseudo-color indicates the average cross-correlation of each voxel with the rest of the brain.

**Figure 3:**
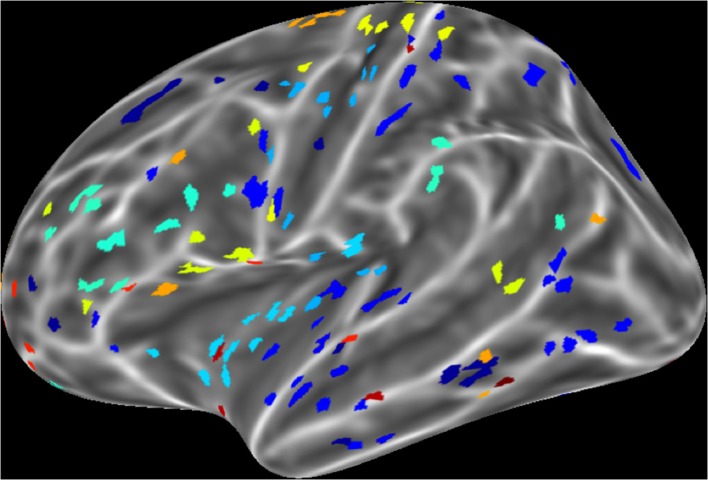
**An example of interacting cortical sites for 10 randomly selected seeds in the model**. Each color indicates a set of 16 sites that interact with the same seed. Interacting sites are chosen based on the 16 highest correlation coefficients of each seed in the all pair-wise correlation matrix.

For a compact illustration of BOLD functional connectivity, the pair-wise correlation values between each site and the rest of the brain were averaged yielding a spatial map of global correlation strength. Figure 1 shows the results for 20 subjects. There is a noticeable variation of connectivity patterns however, the connectivity of a few structures appears to be conserved in most subjects. The global connectivity pattern averaged across all subjects is illustrated in Figure 2. This map emphasizes regions that were most strongly connected with the rest of the brain in all subjects. The average connectivity matrix that gave rise to this figure was used as input data for the simulation. Figure 3 shows an example of the spatial distribution of several sets of 16 interacting sites, i.e., those with the highest correlation with each reference site. Clearly, these interactions reach over large regions of the brain.

### Spin-glass simulation

**Figure 4:**
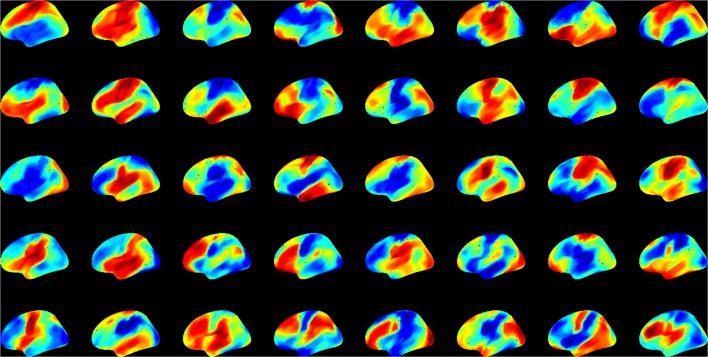
**The 40 most frequent equilibrium patterns at T = 0 activation level from the spin-glass model**. Pseudo-color indicates the probability of UP (red) and DOWN (blue) states of each site (scale is arbitrary). Patterns were classified using hierarchical clustering at 99% similarity after 15 mm spatial smoothing.

First we examined the types of global state patterns that emerged at low activation level T. As anticipated, various metastable state configurations were formed at intermediate T, and they were frozen at low T (Supplementary electronic material). Specifically, at T ≤ 2, the patterns converged, although this sometimes took a long time. To reduce the simulation time to convergence, we performed 10,000 runs at T = 0. This rapidly drove the system to one of many final configurations. We then sought to estimate the diversity of distinct global states. To suppress the effect of local variations, we first performed spatial averaging of each pattern within 5 or 15 mm. The averaging facilitated the comparison of global similarity without local noise. We then used hierarchical clustering of the 10,000 patterns and estimated the relative frequency of patterns. Figure 4 shows the 40 most frequent patterns. All patterns are distinct and they generally reflect known functional regions of the brain (prefrontal, temporal, parietal, occipital, pre- and post-central, etc.).

**Figure 5:**
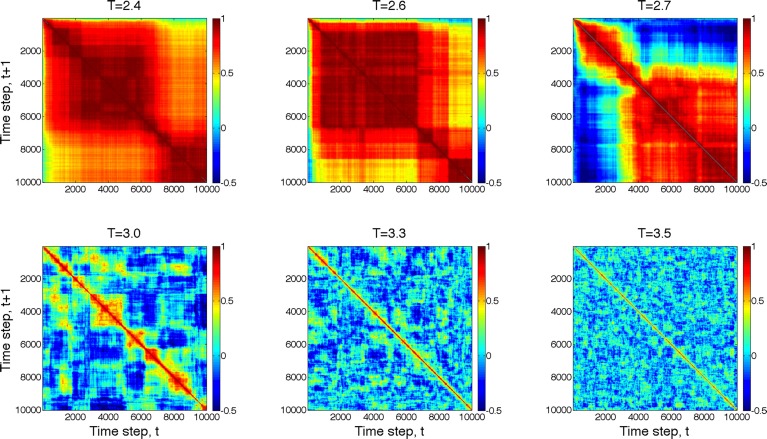
**Temporal correlation of global state patterns at successive time points and different activation levels, T**. Simulation consisted of 10,000 time steps. Pseudo-color indicates the correlation coefficient, cc, of consecutive patterns. Metastable states are formed at T = 2.7 suggesting critical, “edge-of-chaos” behavior. Higher T leads to more random patterns, whereas lower T yields hyper-synchronous, stereotypic patterns.

**Figure 6:**
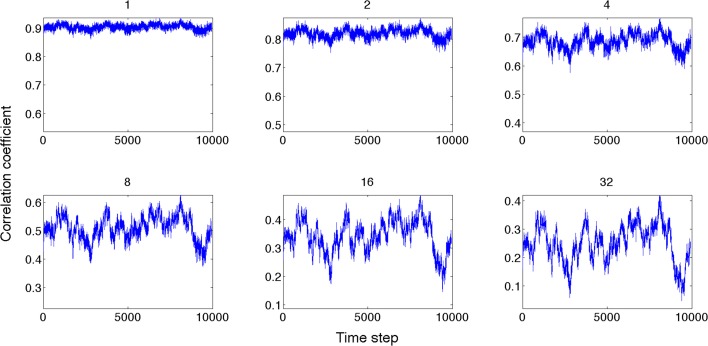
**Global state correlation as a function of time at various time lags (shown on top of each panel)**. Global state patterns become decorrelated at increasing time delays.

**Figure 7:**
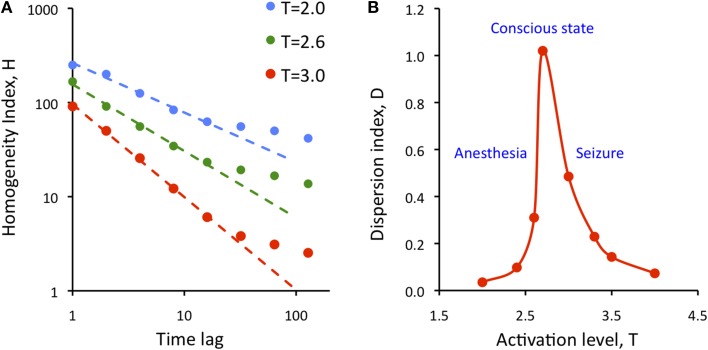
**Dependence of the state repertoire on cortical activation level and embedding time lag**. **(A)** Homogeneity index, H decreases as a function of the time lag of according to power law up to a lag of 20. Decreasing the activation level T (anesthesia) decreases the regression slope. **(B)** Dispersion index, D shows critical behavior as a function of activation level, T. Maximum of D is thought to correspond to the conscious state. D drops at low T (anesthesia) and at high T (seizure).

Next, we compared the dynamics of global state patterns at intermediate activation levels. A convenient measure of the dynamics is the temporal correlation of patterns at successive time points (Figure 5). The correlation matrix at activation level T = 2.7 suggests patterns that are typical of systems with metastable states. At high T, the patterns become random; whereas at low T, the patterns become stereotypic, showing temporal hypersynchrony. Increasing the time lag (embedding delay) from one time step to 2, 4, 8, 16, and 32, reduced the mean and augmented the fluctuation in the correlation of states (Figure 6). This effect was further quantified by the homogeneity index H, defined as the reciprocal of the coefficient of variation:

H = <cc>/SD(cc),

where SD is the standard deviation and brackets <..> indicate averaging. Figure 7A illustrates the results for three levels of activation, T. The H-T relationship followed power law up to a lag of approximately 20 time steps. The power law was preserved at reduced T, although its exponent (the slope of linear regression slope in a log-log plot) was reduced.

To measure the temporal diversity of metastable states, we introduced the *dispersion index* D defined as:

D = K<(1−cc)>/var(cc)

where cc stands for the elements of the all pair-wise correlation matrix of the simulated states, var stands for variance, and K is a normalization constant equal to the variance of the uniform random distribution of the same size as the cc matrix. As defined, D measures the temporal diversity or *repertoire* of global states over time at all time lags. It can be easily seen that the value of D is low for both random and regular systems. Figure 7B shows calculated values of D as a function of activation level T. The plot suggests the presence of typical second-order phase transition. D reaches maximum at T = 2.7; its value drops sharply at both smaller and larger T. Low values of T are thought to characterize suppressed states such as anesthesia or deep sleep, and high values of T are thought to correspond to hyper-activated states, e.g., seizure. At the critical T, metastable states dominate and the diversity of brain states as measured by D is maximum. The high repertoire of states at critical T is consistent with the formerly postulated condition to support conscious cognition.

## Discussion

The goal of this investigation was to demonstrate that long-range neuronal interactions based on empirical measurements in the human brain produce large-scale dynamic patterns of activity. To this end, we applied computer simulation with a modified spin-glass model of site interactions that were constrained by BOLD fMRI functional connectivity. As anticipated, our results predicted large-scale metastable brain states that occurred at an optimal activation level. Simultaneously, at the optimal level of activation, the diversity of state configurations was maximized—consistent with its postulated role in brain functioning in the conscious state. Moreover, the diversity of states was reduced when moving away from criticality—presumably corresponding to states of diminished consciousness.

While a few similar computational studies have been conducted in the past, the present work is novel in several ways. First, the simulation was based specifically on long-range interactions that spanned distances among remote cortical regions. This is the defining difference between the spin-glass model and the Ising model, which considers only nearest neighbor interactions. Second, we used BOLD functional connectivity to select the interacting sites. These empirically-determined connectivity constraints ensure that the model contains connectivity structure similar to that of the actual human brain. Third, we used a novel measure of dispersion to estimate the diversity of global brain states and their dependence on activation level. Therefore, it is of substantial interest that the long-range interacting system, as constrained by real probabilistic data from the brain, readily produced metastable states.

Kitzbichler et al. (2009) demonstrated power law scaling of the synchrony in resting-state fMRI and MEG data, suggesting that the presence of self-organized criticality in the brain is analogous to that obtained from computer simulations on an Ising system. Their simulation was not constrained by actual empirical data. Das et al. (2014) also used the two-dimensional Ising model to illustrate the similarity of measured and simulated fMRI BOLD signals in human subjects. Different from our study, they analyzed BOLD signal distributions above and below threshold to show that the Ising model can predict activity patterns similar to that of BOLD.

Our simulation was different from both of these studies in that ours was based on long-range interactions derived from empirical BOLD functional connectivity. The spatial distribution of interacting sites was determined by the strength of long-range correlations. As a result, the predicted global states resembled large-scale functional patterns of the human brain. Finally, we simulated global brain states at different activation levels.

Another recent simulation study applied the Ising model to fiber-tract data obtained with diffusion tensor imaging (Marinazzo et al., 2014). The outgoing and the incoming information at each network node was quantified as related to the summated input weights and to the time elapsed between consecutive flips of Ising spins. The simulation predicted critical behavior although the profile of state transition was not as rapid as in our simulation.

It could be argued that fiber tract distribution is a more appropriate constraint for the model than functional connectivity. Functional connectivity may not always correspond to direct anatomical connection due to common input or third party interactions. However, a counter argument is that only a fraction of fiber tracts may be used for neuronal communication at any one time, and, therefore, functional connectivity provides a better approximation of the probability of functional interactions regardless of the exact underlying mechanism. Conceivably, real-time measurement of neuronal communication across the whole brain will be the ideal data used as an input to the model when such technology becomes available in the future.

The neurophysiological relevance of the spin-glass model depends on the temporal and spatial scales that it represents. Although the temporal scale of the simulation is arbitrary, it can be grounded in real neuronal events based on empirical data. Spontaneous activity of neuronal populations forms transient spatiotemporal clusters often described as neuronal avalanches (Beggs and Plenz, 2003). The time scale of these events is on the order of 10 ms. Such an alternation between activity and silence of neuronal populations is consistent with the representation of UP and DOWN states of mesoscopic sites in the spin-glass model. On a global spatial scale, EEG topographic maps alternate among microstates at a time scale of approximately 100 ms (Koenig et al., 2002). These states have been linked to fMRI signals (Lehmann, 2010; Musso et al., 2010). The temporal resolution of elementary conscious sensory perception also falls in this order (Bachmann, 2013). The lifetime of the simulated metastable states depends on the chosen level of similarity of the states, i.e., the minimum cross-correlation coefficient at which they are considered equivalent. Accepting a cross-correlation threshold of 0.95, the median lifetime of metastable states at the critical activation level is around 440 ms that is in the timeframe of cognitive phenomena.

We found that the homogeneity index H depended on the embedding time lag according to power law, suggesting scale-free behavior up to a lag of approximately 20 time steps. Based on the preceding considerations, 20 time steps would correspond to approximately 200 ms duration, which agrees with the presumed unit of processing time for conscious computations. Nevertheless, the power law of H does not imply criticality because the power law exponent was close to −1.0 (at T = 3) or less, not −1.5 as previously proposed for critical processes (Beggs and Plenz, 2003). On the other hand, the dispersion index D suggests critical behavior at the phase transition at T = 2.7. D measures the overall diversity of global state configurations across all time points, not only the states' consecutive (or delayed) similarity as H does. At low T, D is small because global brain states are highly correlated and thus (1−cc) is low. Because the sites stochastically fluctuate between UP and DOWN states, the variance of cc does not go to zero and D does not diverge. At high T, D is small again because the variance is large due to the intense random fluctuations. Thus D is high only at intermediate T. The repertoire of global states accessed by the brain over time reaches maximum at a critical point.

During the course of clustering global brain states, a challenge was to define their similarity at a mesoscopic scale with 5000 sites per hemisphere. On one hand, cluster membership had to be defined at a chosen degree of similarity; the number of distinct brain states depended on this choice. We carried out clustering at 99.0 and 99.9% similarity; yielding a higher number of distinct states when the similarity requirement was stricter. Another factor that influenced the clustering was the degree of spatial averaging, for which we used either a 5 or 15 mm radius. This choice made a significant difference at 99.9% similarity but it made very little difference at 99.0% similarity. Guided by these preliminary assessments, we chose 15 mm spatial averaging and 99.0% similarity level for the final simulations.

In this work we sought to gain insight into the possible diversity and dynamics of UP/DOWN state patterns as a measure of complex brain states. It has been hypothesized that dynamic states of connectivity represent modes of cognitive information processing in the brain (Bressler and Menon, 2010). Moreover, the diversity or *repertoire* of brain sates has been postulated as one of the fundamental conditions for information integration in the conscious state or more specifically consciousness itself (Tononi, 2008).

In the spin-glass model, the overall probability of UP/DOWN transitions of mesoscopic sites was controlled by the global activation level T. We saw that at a critical activation level, large-scale metastable states were frequent, and the diversity of global brain states was enhanced. Although this may not be a stable state *in vivo*, approaching criticality may be facilitated by the phasic increases in ascending arousal (Buzsaki et al., 1988), which would repeatedly randomize the system (at high T), and then allow it to sink into new configurations. The latter may have an additional effect on augmenting the repertoire of brain states over time.

If information processing indeed depends on the repertoire of brain states, the question one may ask is how many distinct brain states exist. The answer to this question obviously depends on what we consider the smallest units of the system. The organizational complexity and the number of distinct functional states of the brain would plausibly increase at finer spatial and temporal scales, spanning several hierarchical levels from synapses, neurons, local circuits, to regions and networks. Although the spatiotemporal resolution of fMRI is relatively coarse, the number of combinatorially possible network patterns defined at near voxel level is enormous, and properly sampling these patterns using fMRI is limited. Computer simulation helps extend our ability to estimate the brains state repertoire within empirically set constraints.

An application of interest of the model is examining the effects of general anesthesia, which is characterized by reduced global activation due to a suppression of the ascending arousal system (Nelson et al., 2002; Alkire et al., 2007). As we saw, decreasing the activation level T retards the dynamic transition of global metastable states by reducing the probability of UP/DOWN transitions. As a result, fewer distinct brain states occur over time, which predicts reduced information capacity. As we formerly argued, a reduction in the repertoire of global brain states may underlie anesthetic loss of consciousness (Alkire et al., 2008). An alternative mechanism that may diminish information integration during anesthesia is the weakening of site interactions. This may lead to breakdown of meaningful communication within the brain's critically important functional networks. Although this has not been tested here, the overall effect of reduced connectivity on the global dynamics is expected to be similar to that of reduced cortical arousal. Both mechanisms are likely at work in the mediation of the anesthetic effect.

Although we have emphasized the application to anesthesia, the simulation results equally apply to altered states of consciousness such as deep sleep, vegetative state, coma, or, at the other end of the spectrum, seizure. We saw that both low and high activation levels reduced the diversity of global brain states, presumably pushing the brain away from optimal information processing and integration.

Current views differ on whether critical behavior in the cortex is associated with normal conscious behavior or a transition to altered states of consciousness. Previously, Steyn-Ross et al. (1999) examined first-order phase transitions using a mean-field model of excitatory and inhibitory neuronal groups with relevance to the anesthetic modulation of the state of consciousness. They hypothesized that the anesthetic acted as a randomizing agent to break down the connections between interacting neuronal populations and that this loss of neuronal cooperativity accounted for the loss of consciousness under anesthesia. Alternatively, spontaneous ongoing activity may play a role in inducing state transitions that may be important for maintaining conscious awareness. Recently, Steyn-Ross et al. (2009) suggested that patterns of cortical activation arise from spontaneous self-organization of interacting neuronal populations at a mesoscopic scale. They simulated metastable activation patterns that were altered when the somato-dendritic feedback of neurons was reduced; reflecting a decrease in excitatory neuro-modulation as seen during sleep or anesthesia. We interpret our simulations to be consistent with the ongoing formation of a large diversity of metastable states that are essential for the stream of conscious thought (Werner, 2009). Although the patterns also change spontaneously, fluctuations in the level of cortical activation (cortical arousal) via its randomizing effect may facilitate the rapid formation and transition of consecutive activity patterns, thereby further augmenting the repertoire states accessed by the brain over time.

### Conflict of interest statement

The authors declare that the research was conducted in the absence of any commercial or financial relationships that could be construed as a potential conflict of interest.
